# Editorial: Rising stars in motor neuroscience 2023

**DOI:** 10.3389/fnhum.2024.1380955

**Published:** 2024-02-27

**Authors:** Simone Carozzo, Andrea Demeco

**Affiliations:** ^1^S. Anna Institute – Crotone Italy, Crotone, Italy; ^2^Department of Medicine and Surgery, University of Parma, Parma, Italy

**Keywords:** motor neuroscience, artificial neural networks—ANN, EEG signal analysis, motor process, neuropathic pain (NeP), human neuroscience, rehabilitation, motor disorders

In the ever-evolving landscape of scientific research, it is critical to identify and celebrate emerging talent that will shape the future of their respective fields. Within human neuroscience, the exploration of motor function and its intricate neural mechanisms is essential for understanding human behavior. In this editorial, we shine a spotlight on the rising stars of motor neuroscience, recognizing promising researchers who are poised to revolutionize the field, from the intricacies of motor learning to the rehabilitation of motor disorders.

In recent year, we assisted to a growing enthusiasm in neuroscience, with an increasing number of articles and the introduction of new perspectives in clinical practice. One of the main features, is its transversal nature with various field of application, from neurological patients to sport. In this context, the technological advancement, provided researcher with innovative tools for a deeper understanding of how the nervous system acts in controlling and managing the movement. In this way, the movement analysis goes beyond the classical concept of range of motion and force production, but it provides an insight on the causality of the action.

In rehabilitation, the first step for a personalized program is a detailed and objective evaluation of the patient's deficit. Bongiorno et al. in their study, proposed a protocol coupling kinematic analysis and surface electromyography (sEMG) for the motion analysis of the shoulder in breast cancer patients. The authors highlight the importance of motion analysis in monitoring and optimizing the rehabilitation outcomes, by identifying residual impairments and tracking progress over time to refine treatment strategies and enhance patient care.

However, the amount of the data collected with ever more detailed tools represents a challenge for the researchers. The analysis and, consequently, the interpretation of the signal represent an important topic in clinical research. In this context, Hascher et al. demonstrated the power of multivariate analysis, particularly using artificial neural networks (ANN), compared to traditional univariate and distributed univariate approaches, in identifying EEG correlates of interlimb coupling. By modeling nonlinear relationships between EEG activity and motor movements, multivariate approaches offer valuable insights into the mechanisms underlying motor behaviors, paving the way for personalized analyses and interventions in motor neuroscience.

The role of rehabilitation, in case of lost function, aims to find alternative solutions to improve the patient's quality of life. In this context, brain computer interface could play a key role in the improvement of autonomy in patients with motor impairments. In their study, Lin et al. identified distinct patterns in different frequency ranges during motor imagery. The findings suggest that unilateral LFP signals contain valuable information about arm motor imagery, including laterality and regional specificity, supporting their potential use in brain-computer interfaces for upper limb rehabilitation and neuroprosthetics.

Sports and exercises are beneficial not only for the physical health, but on mental wellbeing. Literature reports a positive impact on cardiovascular and nervous system. At this regard, Chen et al., focusing on postural control in skilled athletes, demonstrate superior postural stability and reduced cortical inhibition during dual-task performance compared to non-athletes. The results of this study emphasize the interconnectedness of cognitive and motor processes in skilled athletes and suggests implications for optimizing training programs in skill-oriented sports.

In spinal cord injuries, neuropathic pain and spasticity are both results from spinal hyperexcitability. In their study, Franz et al. explored the potential use of laser-evoked withdrawal reflexes as an outcome parameter to investigate maladaptive spinal circuitries. Their results suggest that distinct types of maladaptive plasticity may underlie spasticity and neuropathic pain, challenging the assumption of identical underlying mechanisms for these complications. Noxious radiant heat could, therefore, represent a unique assessment to evaluate nociceptive processing.

Moreover, the findings Starkweather et al. challenge the traditional view of somatotopic organization. They present a study on the neural correlates of stereotyped upper and lower limb movements in the human primary motor cortex, highlighting distinct oscillatory signatures for arm and leg movements and suggesting a role for low-frequency oscillations in coordinating movements across different body parts.

In conclusion, the collection of the topic discussed in this Research Topic, are linked by a file rouge represented by the efforts of high-level researcher in providing a breadth and depth analysis in motor neuroscience, ranging from understanding neural mechanisms to developing innovative rehabilitation strategies. They offer valuable insights into the complexities of motor function and pave the way for future advancements in the field.

## Author contributions

SC: Writing—original draft, Writing—review & editing. AD: Writing—review & editing.

